# Comparison of Metabolomic Profiles of Organs in Mice of Different Strains Based on SPME-LC-HRMS

**DOI:** 10.3390/metabo10060255

**Published:** 2020-06-17

**Authors:** Katarzyna Burlikowska, Iga Stryjak, Joanna Bogusiewicz, Bogumiła Kupcewicz, Karol Jaroch, Barbara Bojko

**Affiliations:** 1 Department of Pharmacodynamics and Molecular Pharmacology, Faculty of Pharmacy, Collegium Medicum in Bydgoszcz, Nicolaus Copernicus University, Toruń, dr. A. Jurasza 2 Street, 85–089 Bydgoszcz, Poland; k.burlikowska@cm.umk.pl (K.B.); i.stryjak@cm.umk.pl (I.S.); j.bogusiewicz@cm.umk.pl (J.B.); karol.jaroch@cm.umk.pl (K.J.); 2 Department of Inorganic and Analytical Chemistry, Faculty of Pharmacy, Collegium Medicum in Bydgoszcz, Nicolaus Copernicus University, Toruń, dr. A. Jurasza 2 Street, 85–089 Bydgoszcz, Poland; kupcewicz@cm.umk.pl

**Keywords:** mouse strain, tissue, untargeted metabolomics, metabolomic profile, solid-phase microextraction

## Abstract

Given that the extent to which genetics alters the metabolomic profile of tissues is still poorly understood, the current study aimed to characterize and investigate the metabolite profiles of brain, liver, kidney and skeletal muscle of two common mouse inbred strains (BALB/c, C57BL/6) and one outbred stock (CD1) for strain-specific differences. Male mice (*n* = 15) at the age of 12 weeks were used: BALB/c (*n* = 5), C57BL/6 (*n* = 5) and CD1 (*n* = 5). Solid phase microextraction (SPME) was applied for the extraction of analytes from the tissues. SPME fibers (approximately 0.2 mm in diameter) coated with a biocompatible sorbent (4 mm length of hydrophilic-lipophilic balanced particles) were inserted into each organ immediately after euthanasia. Samples were analyzed using liquid chromatography coupled to a Q-Exactive Focus Orbitrap mass spectrometer. Distinct interstrain differences in the metabolomic patterns of brain and liver tissue were revealed. The metabolome of kidney and muscle tissue in BALB/c mice differed greatly from C57BL/6 and CD1 strains. The main compounds differentiating all the targeted organs were alpha-amino acids, purine nucleotides and fatty acid esters. The results of the study indicate that the baseline metabolome of organs, as well as different metabolic pathways, vary widely among general-purpose models of laboratory mice commonly used in biomedical research.

## 1. Introduction

In view of the resemblance between mice (*Mus musculus*) and humans on genetic, anatomical, physiological, and pathophysiological levels, the mouse is nowadays considered to be the most common model in biomedical research, as well as the most published and well-characterized [1,2]. According to the latest report concerning the number of animals used for scientific purposes in the Member States of the European Union in 2017, mice constituted 61% of all animals used in studies [3]. There currently exists hundreds of established inbred, outbred, and transgenic mouse strains with defined genetic backgrounds and unique features, such as coat color, behavior, metabolism, fertility, immune function, and other physiological traits [4].

Among the most commonly used strains, the inbred C57BL/6 and BALB/c lines, as well as the CD1 outbred stock, are particularly predominant in research. As these strains are considered to be general-purpose models, they are extensively and often interchangeably used in many different disciplines of biomedical research. The first two of the above-mentioned strains are considered ‘classical’ inbred strains, signifying that the animals are genetically homogeneous and individual mice within a strain are identical clones of their parents and siblings [5]. Generally speaking, the use of inbred strains has become standard for research in most areas of mouse biology, as well as for basic and preclinical investigations. Currently, the most common strain, C57BL6, is used in a wide variety of research areas, including cardiovascular and developmental biology, diabetes and obesity, genetics, immunology, neurobiology, and sensorineural research. BALB/c mice are currently among the five most widely used inbred strains in biomedical applications, and a particular favorite in immunology and infectious disease research [6]. CD1 mice, in contrast to the two strains mentioned above, is an outbred stock that is bred specifically to maximize genetic diversity and heterozygosity within a population. The CD1 line is a general multipurpose model that can be used in fields such as toxicology (safety and efficacy testing), aging, and oncology [5,7].

Metabolomics, or metabolic phenotyping, encompasses the analysis of all low-molecular-weight (< 1 kDa) components in a biological sample, and it is generally considered to be a field of research complementary to proteomics, genomics, and transcriptomics. The metabolome, representing the total number of metabolites within a given biological system, may consist of metabolites solely under endogenous control, and may also involve those originating from exogenous sources [8,9]. Sets of small metabolites attainable in metabolomics are closely related to the phenotypes of living organisms and provide information on biochemical activities by reflecting the substrates and products of cellular metabolism [10,11,12]. The metabolome is dynamic and susceptible to many factors such as environment, genetic modifications, changes in gut microflora and altered kinetic activity of enzymes, and can thus provide insights into current cell status as well as describing an actual health condition of the system being studied [13]. One of the analytical chemistry strategies applied in metabolomics is to carry out untargeted analysis of the metabolism so as to investigate the system on a global, relatively unbiased scale, thus aiming to measure the broadest range of metabolites present in an extracted sample without a priori knowledge of the metabolome [14,15]. Owing to its high sensitivity and high throughput, this method has gained wide attention as a method for profiling endogenous metabolites. Untargeted tissue metabolomics in particular can provide valuable insights into the physiological characteristics of the body and greatly enhance our understanding of the biochemistry and metabolism of biological systems [12,16]. However, successful characterization of the metabolome of tissue is often burdened by challenging steps such as tissue collection, quenching of the metabolism, homogenization, and metabolite extraction [17,18].

Solid phase microextraction (SPME) has been proven to be a powerful, low invasive tool for tissue analysis in untargeted metabolomics studies [19], overcoming all the challenges mentioned above while successfully capturing unstable and short-lived metabolites often undetectable via traditional methods. SPME is a relatively young sample preparation method with unique features that enable its successful application in animal studies as an alternative to standard protocols [20,21]. Choices regarding pretreatment methodology play an extremely important role in metabolomic studies since sample pretreatment may affect not only the molecular features available, but also the biological interpretation of the obtained chromatographic data [17]. SPME technology uses special fibers coated with biocompatible sorbents (thin film of polymer) that can be inserted directly into the tissue (brain, muscle, liver, lungs, etc.) in order to extract small molecules in amounts proportional to their biologically active unbound concentrations. SPME has a number of advantages, including simplicity, high sensitivity and a relatively low invasive nature. Moreover, it is a non-exhaustive sample preparation procedure based on chemical biopsy that combines sampling, sample preparation, rapid metabolism quenching, and extraction into a single step [20,21,22,23,24].

Analysis of tissue as a primary site of any dysregulation has been one of the main goals of biomedical experiments using animal models. Therefore, the aim of the present study was to compare the tissue metabolome of selected organs (liver, kidney, brain, and thigh muscle) in the three mouse strains commonly used in biomedical research: C57BL/6, BALB/c and CD1. The outbred stock (CD1) was chosen for this study to find out to what extent an outbred line differs in its metabolome compared to inbred strains. We expect that having an insight into the metabolomic profile of different organs in intact animals will give us an opportunity to find out how much these three popular strains differ among each other in terms of the basic metabolic processes occurring in the body.

## 2. Results

**Figure 1:**
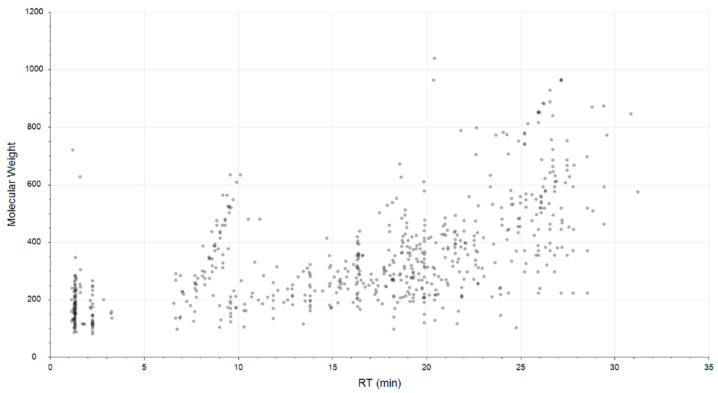
Plot representing the distribution of molecular weight versus retention time (RT) of filtrated features obtained in LC-MS analysis, electrospray ionization in positive ion mode (ESI+). Details of detected features are presented in Table S1.

Figure 1 presents an ion map of molecular features after a data filtration step, whereas an ion map analysis of all molecular features detected in mice tissues is presented in Figure S1.

**Figure 2:**
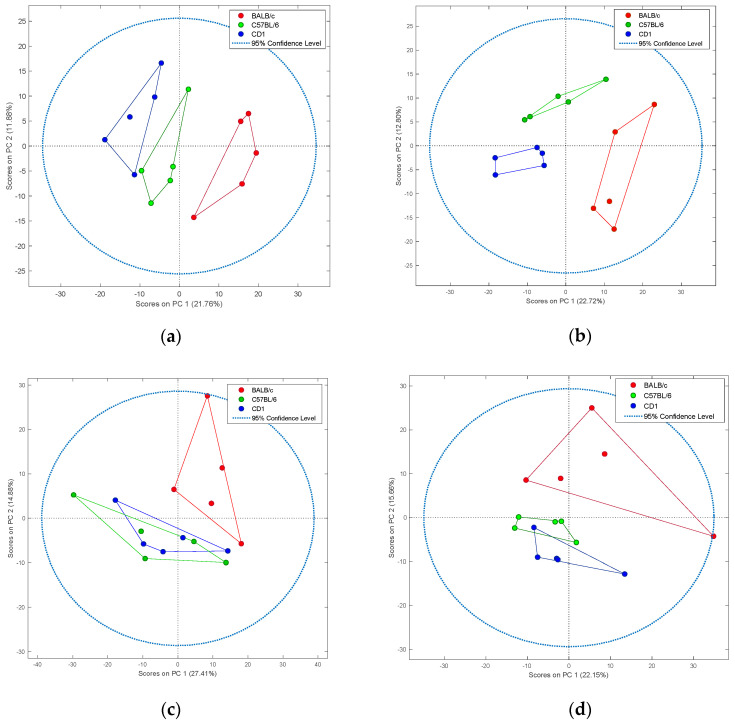
Two-dimensional principal component analysis (PCA) of untargeted metabolomics data of tissues collected from three different mouse strains. Examined strains included: BALB/c (red), C57BL/6 (green), and CD1 (blue). Tissues analyzed included: brain (**a**), liver (**b**), kidney (**c**), and muscle (**d**).

Principal component analysis (PCA) was used to confirm the quality of instrumental analysis (Figure S2) and investigate the differences in the metabolic profiles of organs for all (673) detected features among C57BL/6, BALB/c and CD1 mice. The two-dimensional score plots (PC1 vs. PC2) presented in Figure 2 revealed major differences in metabolomic patterns of brain (Figure 2a) and liver (Figure 2b) tissue between the examined strains. The metabolome of kidney tissue in BALB/c mice distinctly separated from C57BL/6 and CD1 mice, whereas data points in the score plots for C57BL/6 and CD1 mice had mostly overlapping distributions (Figure 2c). Likewise, for muscle tissue, the BALB/c strain separated clearly from C57BL/6 and CD1 mice, whereas distribution differences between C57BL6 and CD1 mice were not so evident (Figure 2d). Clusters were noticeably larger for BALB/c mice in kidney, liver, and muscle tissue, but similar for brain tissue among all examined strains. Additionally, three-dimensional (3D) PCA score plots (representing relationships between PC1, PC2, and PC3) are shown in Figure S3. On the whole, 3D scatter plots revealed similar cluster separations to those of two-dimensional plots. Clear separation of strains within the brain and liver tissues presented in 2D plots was confirmed by three-dimensional visualization. Moreover, many more scattered points for BALB/c compared to the other two mouse strains was also observed.

Identification of detected features was performed on Compound Discoverer 2.1 software, whereas classification of putative metabolites on the basis of their chemical taxonomy was done with the use of Human Metabolome Database HMDB. Selected compounds belonging to a variety of metabolite classes (Table 1) were subjected to analysis of variance (ANOVA), revealing that most of the presented metabolites differentiated (*p* < 0.05) studied strains within a given organ (Table 1). Metabolites differentiating all the examined tissues were found to predominantly belong to alpha-amino acids and derivatives, purine nucleotides and fatty acid ester groups. In addition, metabolomic profiles of brain tissue differed between strains in the levels of metabolites belonging to purine derivatives, purine and pyrimidine nucleosides and derivatives, hydroxy fatty acids and fatty amides, as well as alcohols and polyols. Liver tissue profiles differentiated with respect to levels of purine derivatives, purine nucleosides, ceramides, benzoic acids and derivatives, as well as imidazoles. Benzoic acids and derivatives, as well as N-acyl-alpha-amino acids, differentiated kidney tissues whereas skeletal muscle profiles differed in N-acyl-alpha-amino acids, purine derivatives and alcohol and polyol levels.

**Figure 3:**
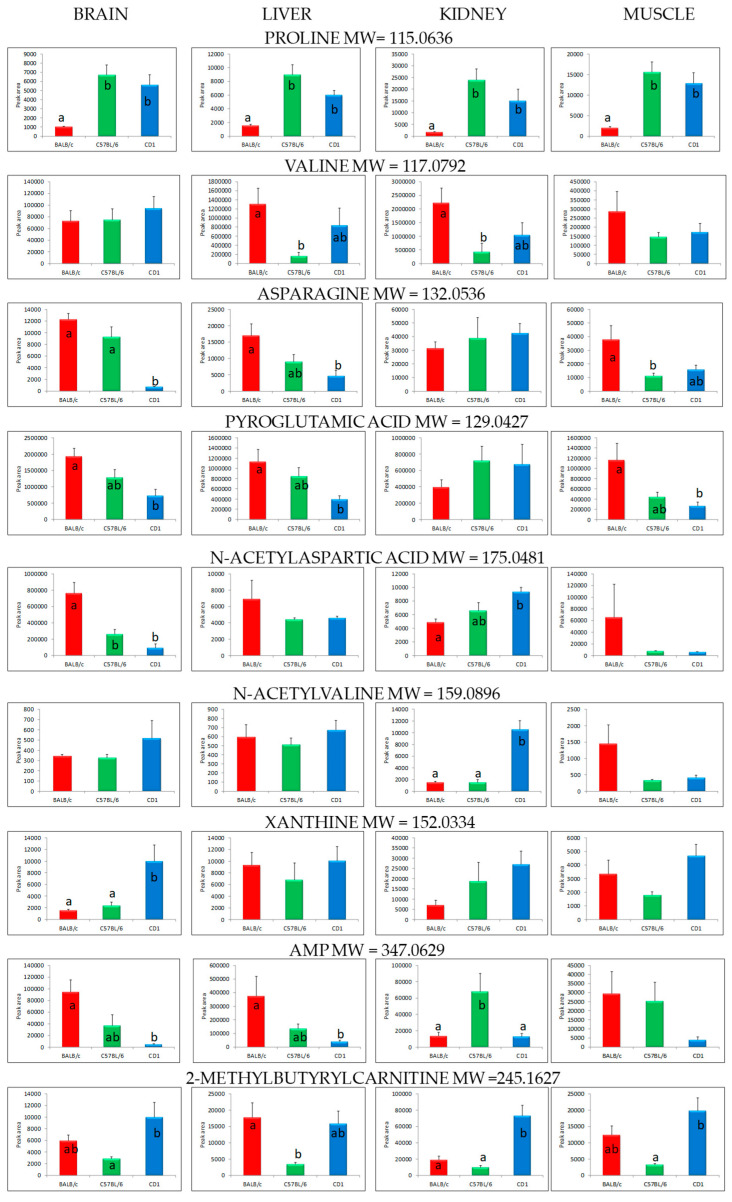
Levels of selected metabolites present in brain, kidney, liver and muscle tissues of BALB/c, C57BL/6 and CD1 mice. Data is presented as mean ± standard error of the mean (bar: $\bar{x}$; whisker- standard error of $\bar{x}$); a, b: bars with different letters differ significantly at *p* < 0.05 (post-hoc Tukey HSD test); MW: molecular weight.

Table 1 also contains compounds that did not differentiate the targeted organs (*p* > 0.05) but were introduced for further comparative analysis. Figure 3 presents the differences in the levels of metabolites that were common among all tissues.

**Figure 4:**
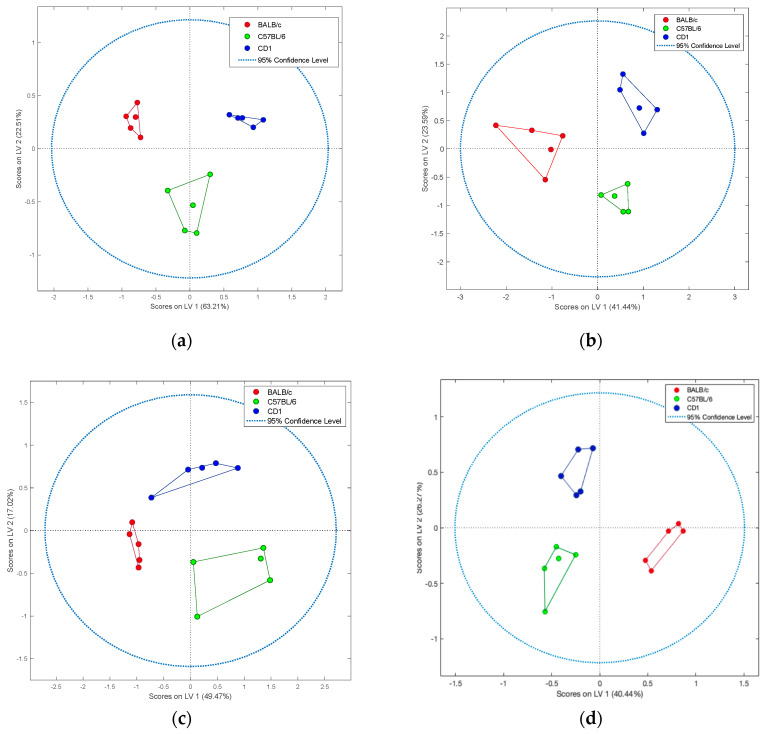
Score plots (PLS-DA) of selected metabolites in tissues collected from three different mouse strains. Examined strains included: BALB/c (red), C57BL/6 (green), and CD1 (blue). Tissues analyzed included: brain (**a**), liver (**b**), kidney (**c**), and muscle (**d**).

The selected metabolites listed in Table 1 were additionally subjected to the partial least squares discriminant analysis (PLS-DA). Results are presented in Figure 4 (scores plots), Figure S4 (loading plots) and Table S2 (validation metrics).

The PLS-DA method was used to more specifically model differences in the metabolome profiles of the targeted tissues of the compared strains of mice. Each model was validated (venetian blinds cross-validation) and refined using a permutation test (number of permutations = 100). This statistical analysis refined the number of features, yielding an optimal set of compounds from the selected metabolites (presented in Table 1) that successfully differentiated strains within each organ. For brain and liver tissues, the distinctions observed among BALB/c, C57BL/6 and CD1 mice were mainly similar to the results obtained in the PCA; however, for kidney and muscle, the separation between groups was much cleaner, as typically expected from supervised chemometric methods.

**Figure 5:**
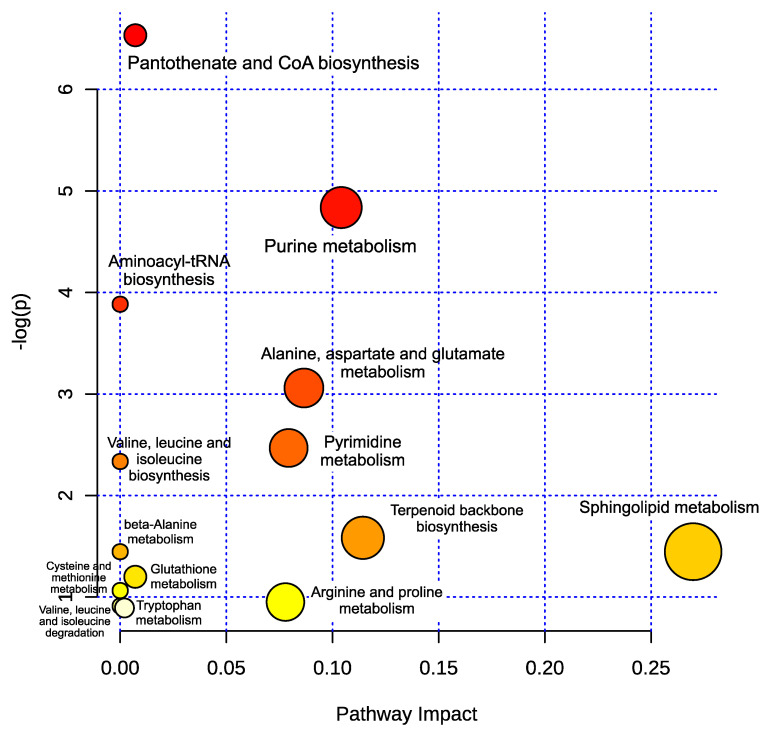
Pathway analysis of compounds differentiating examined mice strains. The *Y*-axis represents log *p*-values obtained from the pathway enrichment analysis. The *X*-axis represents pathway impact values obtained from pathway topology analysis. Node colors and radii are based on *p*-values and pathway impact values, respectively.

A metabolic pathway analysis was carried out to investigate the key biochemical pathways of the selected metabolites (Table 1). The results from the pathway analysis are shown graphically in Figure 5, while the compounds involved in the respective metabolic pathways are presented in Table S3. The analysis uncovered a total of fourteen metabolic pathways mainly involved in the metabolism of amino acids (beta-alanine, valine, leucine, isoleucine, cysteine, methionine, tryptophan, arginine, proline, asparagine, glutamine), the biosynthesis of the aminoacyl-tRNA, terpenoid backbone, pantothenate and CoA, as well as the metabolism of glutathione, sphingolipids, pyrimidine, and purine.

## 3. Discussion

Mammalian tissues, representing the sites of cellular metabolism, are a main target for metabolomics experiments, which can give us a ‘snapshot’ of a given tissue’s temporary state when properly performed [25]. Considering that the extent to which genetics alters metabolism at the tissue level is still poorly understood, we conducted this study to find out whether two common mouse inbred strains (BALB/c, C57BL/6) and an outbred stock (CD1) are characterized by peculiar, strain-specific metabolite profiles, considering the selected organs (brain, liver, kidney and skeletal muscle) as optimal sites of extraction given their predominant use in research. The chosen lines of mice are general multipurpose models that have been used interchangeably for different scientific purposes. This particularly applies to the two inbred strains (BALB/c and C57BL/6) selected in this study. Inbred mice are defined as genetically identical within the strain and are used in studies in which isogenicity and homozygosity in the test population are desired. Conversely, in an outbred mouse stock (CD1), each animal is genetically unique and phenotypic variation in outbred stocks is usually greater than that of inbred strains due to both genetic and non-genetic factors [5].

Bearing in mind the fact that an individual’s metabolome is sensitive to many internal and external variables, including age, gender, diet, environment, time of day, and even one’s own genetics [12], the present study was performed on animals of the same age and sex that were kept in standard environmental conditions. Furthermore, we used intact mice (no experimental factors were applied) so we could compare the metabolomic profile of mouse organs under physiological conditions. The SPME technique was utilized as a sampling tool as it has been proven to be well suited for metabolomic analysis of tissue [19,20,21]. SPME additionally enabled extractions immediately after organ collection in our experiments, which is particularly desirable in rodent studies given that the metabolomic profile of tissues changes rapidly after cessation of blood circulation [25].

The PCA clearly revealed distinct differences in the entire metabolome of the brain and liver among all examined strains. The kidney and muscle metabolomic profiles of BALB/c mice distinctly varied from C57BL/6 and CD1, whereas these profiles in C57BL/6 and CD1 strains were, to some extent, similar. It should be stressed that the size of the clusters was larger for BALB/c in liver, kidney and muscle tissues, indicating greater variability in the metabolome, whereas in brain tissue, cluster size was similar for all the examined strains. In a previous study aimed at profiling metabolites in brain, heart, kidney, and liver tissues of 26 mammalian species representing ten taxonomical orders, it was suggested that brain metabolites are the most conserved among the examined organs, and have evolved largely according to the phylogeny. In contrast, the metabolites of other examined organs diverged to a much greater extent, possibly due to stronger environmental influences or other selection pressures [26]. Previous reports [27,28] clearly indicate that genetic background has profound effects on the overall metabolite profiles of murine tissues, a factor that was confirmed in our experiment, especially for the brain and liver. It is also worth mentioning that significant phenotypic differences covering a number of physiological, biochemical, and neurobehavioral systems have been previously identified, even between very close mouse strains such as C57BL/6J and C57BL/6N [27].

In our work, amino acids and derivatives turned out to be the main group of compounds differentiating strains within individual organs. Considering brain tissue, the level of proline was significantly higher in C57BL/6 and CD1 mice compared to BALB/c. It must be stressed that a similar trend was also observed for all other examined organs. Proline is an endogenous amino acid in mammals that plays an essential role in primary metabolism and physiologic functions of living organisms. It can be endogenously synthesized either from glutamate or ornithine. Proline plays an important role in the synthesis and structure of proteins and in their metabolism (particularly the synthesis of arginine, polyamines, and glutamate via P5C), and can act as a direct substrate for ATP production [29,30]. It has also been revealed that, under certain conditions, proline present in the brain can act as a neurotoxin that non-selectively destroys pyramidal and granule cells in rats [31]. The BALB/c strain had the lowest level of brain proline but was characterized by the highest levels of other amino acids such as asparagine, pyroglutamic acid, and N-acetylaspartic acid. These differences were significant compared to the levels of these metabolites in brain tissue of CD1 mice.

N-acetylaspartic acid (NAA), one of the most concentrated molecules in the central nervous system, is synthesized from aspartate and acetyl-coenzyme A in neurons. Its metabolic and neurochemical functions are still under investigation, but it is suggested that NAA is a direct precursor for the synthesis of the important dipeptide neurotransmitter N-acetylaspartylglutamate. It plays a role in neuronal osmoregulation and axon–glial signaling as well as in brain nitrogen balance [32]. Studies on mice and rats have shown that brain N-acetylaspartic acid concentrations remain relatively constant in different strains [33]. Considering mice, only two strains (DBA 2J/Sel and C57 BC/cdJ/Sel) had slightly higher levels of N-acetylaspartic than the other strains tested. We compared quite different strains, among which BALB/c mice were characterized by the highest content of this metabolite in the brain compared to C57BL/6 and CD1.

Valine, along with leucine and isoleucine, belongs to the branched chain amino acid (BCAA) group which cannot be synthesized de novo in mammals and must be supplied by a diet. In the central nervous system, BCAAs play a crucial role by providing nitrogen for the synthesis of the neurotransmitter glutamate [29]. In the present study, the level of valine in brain tissue did not differ significantly among the tested strains of mice. Previous research on (NIH) Swiss mice revealed that the levels of branched chain aliphatic amino acids (leucine, isoleucine, allo-isoleucine, and valine) detected in brain tissue were relatively low compared to other amino acids [34]. The same study showed that the most prevalent of all free brain amino acids was glutamic acid, followed by glutamine and aspartic acid. The present study did not show variability in the levels of the above mentioned brain amino acids, but we found differences in the levels of derivatives such as pyroglutamic acid (present in substantial amounts in the brain and other mammalian tissues [35]) and N-acetylaspartic acid. The highest levels of these amino acids were found in the brain tissue of BALB/c mice, whereas the lowest levels were present in CD1 mice, which suggests strain differences in the metabolism of glutathione and the neurotransmitter glutamate.

The results of our study suggest that purines (nucleotides, nucleosides and derivatives) are also among the main metabolites differentiating strains at the tissue level. They are crucial compounds for cell life. They are coenzymes, sources of energy, and direct precursors of DNA and RNA; moreover, they are involved in many other important biological processes [36]. A comparison of brain tissue in five different strains, including C57BL/6J and BALB/c, revealed that adenosine levels measured at basal conditions significantly varied with respect to mouse strain. Generally, levels of adenosine in the brain have been found to be low, with the exception of the BALB/c strain, which presented relatively higher adenosine levels [37]. In our study, the levels of adenosine monophosphate (AMP) (a direct precursor of adenosine in cells and a second messenger in many biological processes) detected in the brain tissue of BALB/c mice were higher compared to C57BL/6 and CD1 mice, which corresponds with the findings cited above. At the same time, levels of xanthine, an intermediate in the degradation of adenosine monophosphate to uric acid, were highest in the brain of CD1 mice in comparison to the other two mouse strains, which testifies to interstrain differences in purine metabolism.

Our research showed that in addition to amino acids and purines, compounds such as fatty acid esters, ceramides, benzoic acids, and imidazoles differentiated the liver tissue of the studied strains. Detected levels of valine, asparagine, pyroglutamic acid, AMP and 2-methylbutyrylcarnitine were highest in the liver tissue of BALB/c mice among the compared strains, thereby pointing to differences in relevant metabolic pathways (biosynthesis of panthotenate and CoA, aminoacyl-tRNA, biosynthesis and degradation of valine, leucine, and isoleucine, as well as metabolism of purines, glutathione, alanine, aspartate and glutamate) compared to C57BL/6 and CD1 strains. We did not see significant differences in the levels of all these metabolites between C57BL/6 and CD1 mice. Previous reports [26] have revealed that liver tissue is rich in a wide range of metabolites, including amino acids, glycerophospholipids, carbohydrates, and steroids likely indicative of liver-specific pathways. Moreover, it has been proven that the underlying metabolome of liver tissue in mice is highly sensitive to genetic differences, which is in agreement with the results of our study. A previous comparison of different mouse strains, among which C57BL/6J was present, showed that levels of metabolites involved in purine and pyrimidine metabolism, as well as pathways that play a role in amino acid metabolism in the liver, presented significant differences with respect to strain [28]. Although different mouse lines were compared in this study, amino acids, purine nucleotides, nucleosides and purine derivatives were among the main classes differentiating liver tissue. We also found that liver was the only tissue in which interstrain differences in allantoin levels were demonstrated. Allantoin and uric acid are the key compounds of purine nucleotide catabolism, which are formed in the liver as well as many other organs in rats [36].

The present study also revealed that strain type exerted a large effect on the selected metabolites of kidney tissue. We found interstrain differences in the levels of some amino acids, as well as in the levels of purines, fatty acids esters, and benzoic acids. The highest levels of valine and the lowest levels of proline, N-acetylasprtic acid, N-acetylvaline, AMP, and 2-methylbutyrylcarnitine were found in the kidney tissue of BALB/c mice, which generally presented a metabolome that differed considerably compared to C57BL/6 and CD1 mice. Ma et al. [26] found that most of the detected proteinogenic amino acids were present at moderate to high levels in kidney tissue relative to other organs, which can be interpreted as evidence that the metabolite profile of an organ reflects its biological functions.

Our results for skeletal muscle analyses revealed that strain affected the levels of some amino acids. The highest levels of asparagine and pyroglutamic acid and the lowest level of proline were typical of BALB/c, which according to our PCA analysis, separated best compared to C57BL/6 and CD1 mice. Interstrain differences were also found in the levels of pantothenic acid, a water-soluble vitamin; in mice, this compound plays a vital role in the growth of juveniles and in muscle maintenance of adults, as it regulates proper muscle mass among other functions [38]. A pathway analysis revealed that the metabolites differentiating the strains were involved in the metabolism of amino acids (arginine and proline) as well as in the biosynthesis of panthothenate, CoA, and aminoacyl-tRNA. Our results are in agreement with the previous research, which also demonstrated that strain affected the metabolomic profiles of skeletal muscle and pathways involved in energy metabolism (pantothenate and CoA biosynthesis and TCA cycle) in mice [28]. The same study proved that the examined tissues (muscle and liver) were largely unaffected by sex, suggesting that the tissue metabolome remains largely stable across sex.

## 4. Materials and Methods

### 4.1. Chemicals

External calibrant Pierce LTQ Velos ESI Positive Ion Calibration Solution was purchased from Thermo Scientific. All other chemicals were purchased from Sigma Aldrich (Poznan, Poland). Isopropanol, methanol, water, acetonitrile and formic acid were LC-MS grade. For SPME fiber preparation, N,N-dimethylformamide American Chemical Society grade (ACS) reagent and polyacrylonitrile were used.

### 4.2. Materials

SPME fibers were manufactured in house, as described by Gomez-Rios et al. [39]. Probes with a 4 mm extractive phase coating were manufactured with the use of 5 μm hydrophilic-lipophilic balanced (HLB) particles provided by Waters (Wilmslow, U.K.).

### 4.3. Animal Handling and Tissue Collection

BALB/c and C57BL/6 mice were purchased from the Experimental Medicine Centre of the Medical University in Bialystok, while CD1 mice were purchased from Jagiellonian University Medical College, Krakow. Experiments were performed on 15 adult males (aged 12 weeks) for a total of five mice per strain: BALB/c, C57BL/6 and CD1. The animals were housed in a controlled environment: temp. 22 ± 2 °C, 12 h light–dark cycle, humidity 55 ± 10%, with standard mouse chow and water available ad libitum. The mice were sacrificed by manual cervical dislocation, which resulted in euthanasia within approximately 10 s. Once euthanasia was confirmed, brain, liver, kidney and thigh muscle were immediately collected. According to European Union law, permission from the Local Ethical Commission is not required for the use of animal tissue or organs for scientific purposes.

### 4.4. Solid Phase Procedure and Sample Preparation

SPME fibers coated with a biocompatible sorbent (4 mm length of HLB extraction phase) were used for the extraction of metabolites from the selected tissues. Before sampling, all fibers were conditioned overnight with methanol:water, 1:1, *v*/*v* solution. Prior to each extraction, fibers were rinsed for a few seconds in purified water to remove residues of organic solvents. A total of two fibers were inserted per organ for an extraction period of 15 min. Immediately after sampling, fibers were removed from the organ, quickly rinsed with water, then gently dried with wipes to remove any residue of the examined tissue and blood. Afterwards, fibers were placed into empty 0.3 mL polypropylene vials and stored in a freezer at −30 °C until analysis. Desorption was concurrently performed for all fibers directly before instrumental analysis. SPME fibers were placed into vials containing 200 µL of desorption solution consisting of acetonitrile:water (80:20, *v*/*v*) for 120 min with simultaneous vortex agitation (1500 rpm).

### 4.5. Liquid Chromatography–High Resolution Mass Spectrometry Analysis (LC–HRMS)

Samples were analyzed using a LC-HRMS procedure on an ultra-high performance liquid chromatograph coupled to a Q-Exactive Focus Orbitrap mass spectrometer. An instrumental method was adopted from Vukovic et al. [40]. Analytes were separated using a pentafluorophenyl column (Supelco Discovery HS F5, 2.1 mm × 100 mm, 3 μm). Phase A was water + 0.1% formic acid and phase B was acentonitrile + 0.1% formic acid. The gradient was as follows: 0–3 min 0% B, 3–25 min linear gradient to 90% B, 25–34 min 90% B, 34–40 min 0% B. Flow was set to 0.3 mL/min and injection volume was 10 μL. The column temperature was set to 25 °C and sample vials were held at 4 °C in the autosampler.

Mass spectrometer parameters in positive ionization mode were as follows: sheath gas flow rate: 40 a.u.; aux gas flow rate: 15 a.u.; spare gas flow rate: 0 a.u.; spray voltage 1.5 kV; capillary temp 300 °C; aux gas heater temp 300 °C, S-lens radio frequency (RF) level 55; S-lens voltage 25 V; skimmer voltage 15 V. Scan range was set on *m/z* 80–1000 with resolution 70,000. Acquisition was performed using automatic gain control (AGC) target 1E6 and inject time to C-trap was set on auto. The instrument was calibrated using external calibration every 72 h, resulting in mass accuracy <2 ppm. Within-sequence samples were randomized. Pooled quality control (QC) samples composed of 10 µL of each sample were run every 12 injections to monitor instrument performance.

The structure of selected compounds was confirmed based on suitable LC retention time and <3 ppm mass accuracy. Full MS/dd-MS2 confirmation mode was used for this purpose. Fragmentation parameters were as follows. Mass resolution: 35,000 full width at half maximum (FWHM), AGC target: 2E4, minimum AGC: 8E3, intensity threshold: auto, maximum IT: auto, isolation window: 3.0 *m/z*, stepped collision energy: 10 V, 20 V, 40 V, loop count: 2, dynamic exclusion: auto.

### 4.6. Data Processing and Statistical Analysis

Raw MS data was processed by Compound Discoverer 2.1 (Thermo Fisher Scientific) to putatively identify metabolites. QC-based area correction was set to min 50% QC coverage and max QC area relative standard deviation (RSD) 30%. Only features with min peak intensity 105 and signal to noise ratio > 3 were taken into consideration. A data filtration step removed 8795 (93%) of the 9468 features. Putative identification of features was performed by searching for the exact molecular weights of identified features (3 ppm accuracy) in the Human Metabolome Database (HMDB) and Kyoto Encyclopedia of Genes and Genomes (KEGG) online databases. Annotations were verified according to the isotopic distribution of molecules using the ChemSpider Database as a reference (Table S4). The MetaboAnalyst 4.0 software and the KEGG pathway library corresponding to the *Mus musculus* metabolome were used to obtain metabolic pathways associated with significantly differential metabolites. Identification of spectra of fragmented compounds was done with the use of Thermo Scientific FreeStyle 1.4 software linked to online mzCloud database (Table S4).

The averaged peak areas (from two independent SPME fiber replicates) for the obtained compounds were analyzed using Statistica 13.3 PL software (StatSoft, Inc., Tulsa, Oklahoma, USA) via one-way analysis of variance (ANOVA). A post-hoc Tukey Honestly Significant Difference (HSD) test was used to determine the significance of differences among groups, where a *p*-value of < 0.05 was considered to be significant. Resulting data were then exported to the (PLS)-Toolbox (Eigenvector Research Inc.) in Matlab^®^ version 2018b (MathWorks Inc., Natick, MA, USA) for multivariate statistical analysis. Data was log-transformed and mean-centered prior to principal component analysis (PCA) and partial least squares discriminant analysis (PLS-DA). The PLS-DA model was cross-validated using the venetian blinds method and refined by random permutation (100 times) of the Y variable. Two-dimensional score plots were generated to visually assess separation between sample groups.

## 5. Conclusions

The results of the present study clearly indicate that the baseline metabolomic profiles of organs, especially brain and liver tissue, as well as different metabolic pathways, vary widely among the laboratory mouse strains commonly used in biomedical research. Interstrain differences in the metabolome at the tissue level testify that even general-purpose models can give different answers to the same experimental factors, and as a result, yield contradictory outcomes in a study if such factors are not accounted for. For this reason, close attention should be paid when choosing a mouse strain for a particular purpose of scientific research, and considerations of strain variability should also be included in the interpretation of results. Further, the present study corroborates that SPME is an easy, quick and reliable sample preparation method that is ideally suited for tissue analysis in metabolomic studies. However, one needs to remember that the simplicity of the method compromises coverage of analytes compared to multi-step and time-consuming liquid–liquid extraction, and for in-depth investigation of all metabolites, a traditional approach should be considered. Also, it must be pointed out that our results are based mainly on the putative identification of metabolites, so further LC–MS/MS analysis with a high level of confidence is still required to confirm the identities of all metabolites.

## Figures and Tables

**Table 1:** Chemical taxonomy of selected annotated features present in examined tissues of BALB/c, C67BL/6 and CD1 mice.

Organ	Class	Compound	Molecular Weight	Retention Time (min)	*p*-Value (ANOVA)
Brain	Alpha-amino acids and derivatives	Proline	115.0636	1.80	0.0019
		Valine	117.0792	1.28	0.6876
		Asparagine	132.0536	1.19	0.0000
		Pyroglutamic acid	129.0427	1.22	0.0104
		Cystine	240.0237	1.17	0.0441
		N-acetylaspartic acid	175.0481	2.12	0.0005
		Tetrahydrodipicolinate	171.0532	1.36	0.0145
	N-acyl-alpha-amino acids	N-acetylvaline	159.0896	7.69	0.3731
	Purine derivatives	Xanthine	152.0334	3.23	0.0065
	Purine nucleotides	Adenosine monophosphate (AMP)	347.0629	1.34	0.0063
	Purine nucleosides	Inosine	268.0807	6.68	0.0070
		8-hydroxydeoxyguanosine	283.0916	6.93	0.0005
	Pyrimidine derivatives	Uracil	112.0276	1.34	0.0091
	Pyrimidine nucleosides	2’-deoxycytidine	227.0905	7.05	0.0002
	Fatty acid esters	2-methylbutyrylcarnitine	245.1627	17.87	0.0277
	Hydroxy fatty acids	Mevalonic acid	148.0737	1.33	0.0483
	Fatty amides	Oleamide	281.2718	20.64	0.0079
	Alcohols and polyols	Pantothenic acid	219.1107	7.08	0.0273
Liver	Alpha-amino acids and derivatives	Proline	115.0636	1.80	0.0005
		Valine	117.0792	1.28	0.0466
		Asparagine	132.0536	1.19	0.0190
		Pyroglutamic acid	129.0427	1.22	0.0083
		Tetrahydrodipicolinate	171.0532	1.36	0.0185
		N-acetylaspartic acid	175.0481	2.12	0.5917
	N-acyl-alpha-amino acids	N-acetylvaline	159.0896	7.69	0.3605
	Purine derivatives	Xanthine	152.0334	3.23	0.6444
		5-hydroxyisourate	184.0233	1.34	0.0004
	Purine nucleotides	Adenosine monophosphate (AMP)	347.0629	1.34	0.0490
	Purine nucleosides	Inosine	268.0807	6.68	0.0120
		8-hydroxydeoxyguanosine	283.0916	6.93	0.0033
	Fatty acid esters	2-methylbutyrylcarnitine	245.1627	17.87	0.0235
		Ethyl eicosapentaenoic acid	330.2557	21.65	0.0148
	Ceramides	Ceramide (d40:1)	621.6063	26.30	0.0175
	Benzoic acids and derivatives	2-aminobenzoic acid	137.0476	1.33	0.0048
	Imidazoles	Allantoin	158.0439	1.15	0.0442
Kidney	Alpha-amino acids and derivatives	Proline	115.0636	1.80	0.0069
		Valine	117.0792	1.28	0.0446
		Asparagine	132.0536	1.19	0.7313
		Pyroglutamic acid	129.0427	1.22	0.4256
		N-acetylaspartic acid	175.0481	2.12	0.0080
	N-acyl-alpha-amino acids	N-acetylvaline	159.0896	7.69	0.0000
	Purine derivatives	Xanthine	152.0334	3.23	0.1554
	Purine nucleotides	Adenosine monophosphate (AMP)	347.0629	1.34	0.0176
	Fatty acid esters	2-methylbutyrylcarnitine	245.1627	17.87	0.0003
		Ethyl eicosapentaenoic acid	330.2557	21.65	0.0066
	Benzoic acids and derivatives	2-aminobenzoic acid	137.0476	1.33	0.0067
Muscle	Alpha-amino acids and derivatives	Proline	115.0636	1.80	0.0015
		Valine	117.0792	1.28	0.3540
		Asparagine	132.0536	1.19	0.0236
		Pyroglutamic acid	129.0427	1.22	0.0206
		N-acetylaspartic acid	175.0481	2.12	0.3746
	N-acyl-alpha-amino acids	N-acetylvaline	159.0896	7.69	0.6755
		N-tridecanoylglycine	271.2147	22.52	0.0407
	Purine derivatives	Xanthine	152.0334	3.23	0.0635
		5-hydroxyisourate	184.0233	1.34	0.0038
	Purine nucleotides	Adenosine monophosphate (AMP)	347.0629	1.34	0.1549
	Fatty acid esters	2-methylbutyrylcarnitine	245.1627	17.87	0.0051
	Alcohols and polyols	Pantothenic acid	219.1107	7.08	0.0305

## Supplementary Materials

The following are available online at https://www.mdpi.com/2218-1989/10/6/255/s1, Figure S1: Ion map (molecular weight versus retention time) of all extracted metabolites following LC-MS analysis in positive ionization mode, Figure S2: Principal component analysis (PCA) plot of all analyzed samples and extraction quality control (QC) samples, Figure S3: 3D PCA score plots of untargeted metabolomics data of tissues collected from three different mouse strains. Examined strains included: BALB/c (red), C57BL/6 (green), and CD1 (blue). Tissues analyzed included: brain (a), liver (b), kidney (c), and muscle (d), Figure S4: Loadings plots of PLS-DA. Tissues analyzed were: brain (a), liver (b), kidney (c), muscle (d), Table S1: Details of detected features, Table S2: Validation metrics of PLS-DA models, Table S3: Metabolic pathways and involved metabolites differentiating tissues of C57BL/6, BALB/c and CD1 mice strains, Table S4: Isotope pattern matching analysis of differential metabolites using the ChemSpider database.
