# Supplementary material for: Comparison of Metabolomic Profiles of Organs in Mice of Different Strains Based on SPME-LC-HRMS
Source: Metabolites. 2020 Jun 17;10(6):255. doi: 10.3390/metabo10060255 (PMC7345432; doi:10.3390/metabo10060255)
Supplement: Supplementary file 1 [file metabolites-10-00255-s001.pdf]

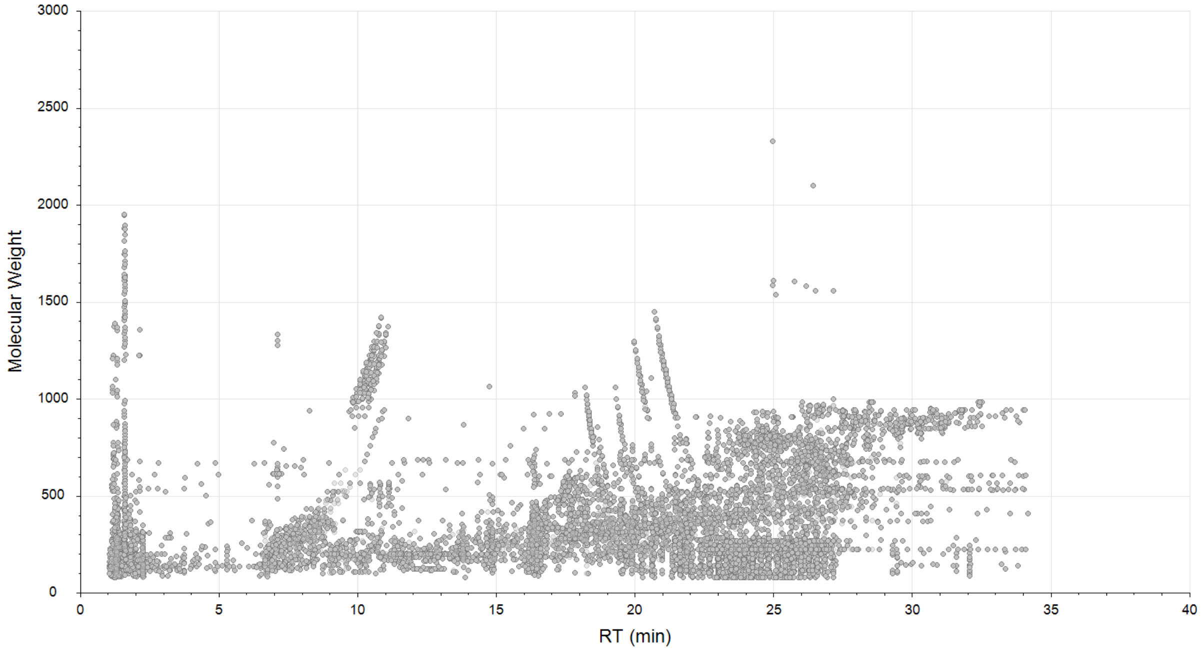

Figure S1. Ion map (molecular weight versus retention time) of all extracted metabolites following LC-MS analysis in positive ionization mode.

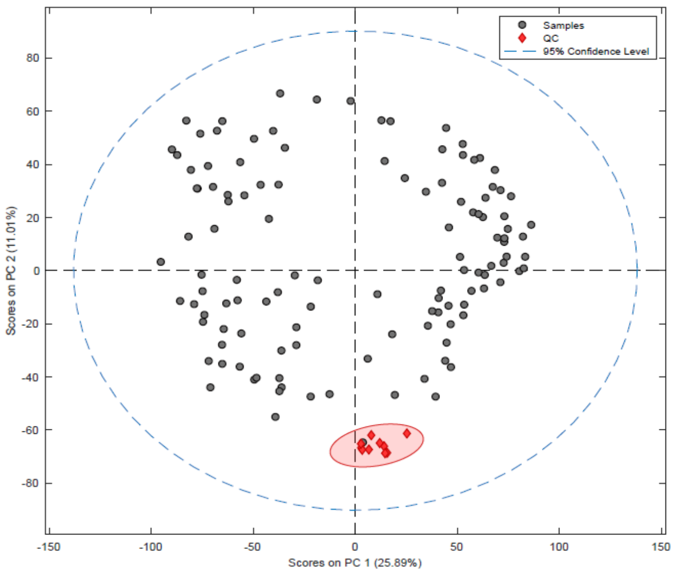

Figure S2. Principal component analysis (PCA) plot of all analyzed samples and extraction quality control (QC) samples.

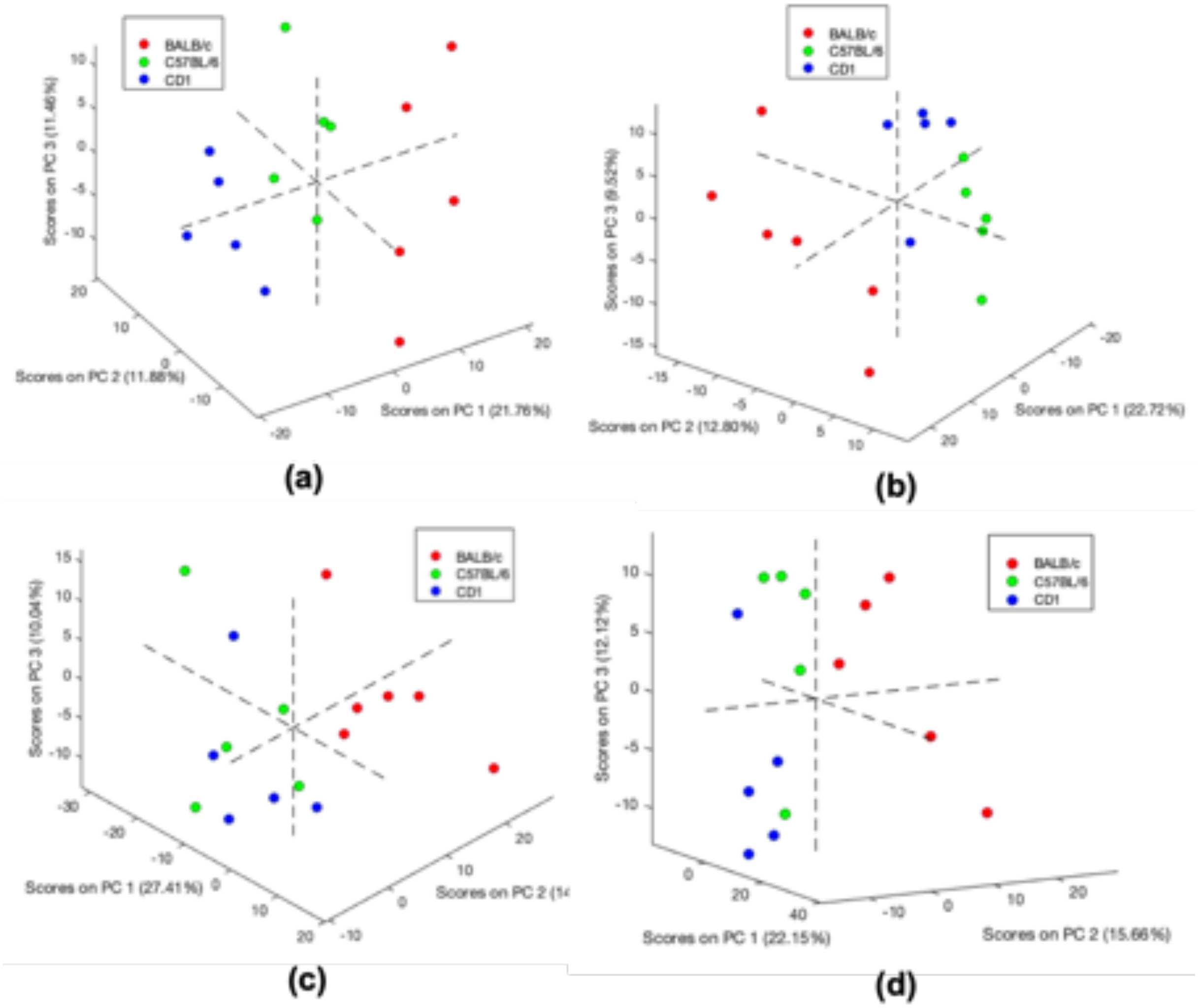

Figure S3. 3D PCA score plots of untargeted metabolomics data of tissues collected from three different mouse strains. Examined strains included: BALB/c (red), C57BL/6 (green), and CD1 (blue). Tissues analyzed included: brain (**a**), liver (**b**), kidney (**c**), and muscle (**d**).

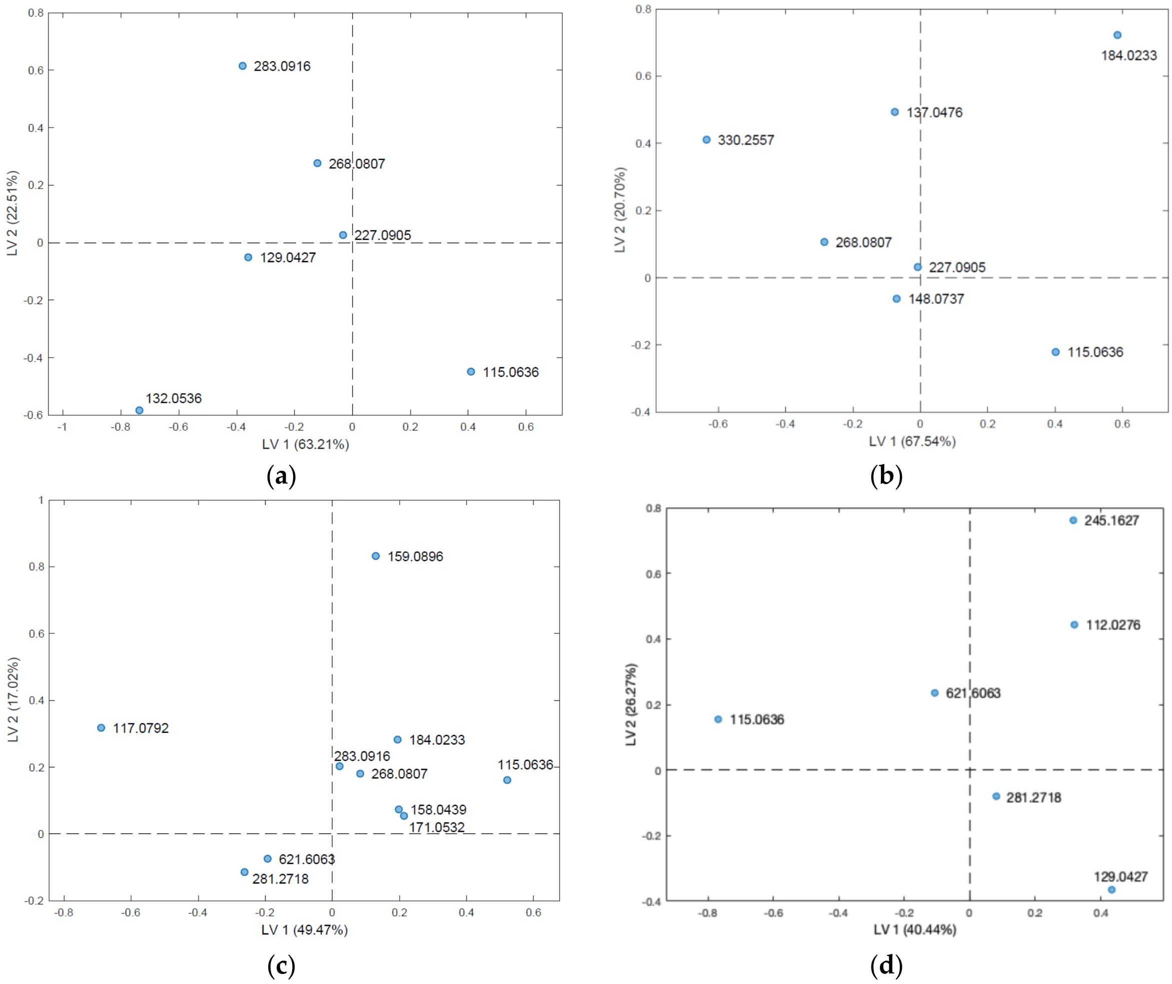

Figure S4. Loadings plots of PLS-DA. Tissues analyzed were: brain (**a**), liver (**b**), kidney (**c**), muscle (**d**).

Table S1. The list of detected features

m/z	Adduct	Polarity	Charge State	Retention time
81.5212	M+H	+	1	2.25
88.0237	M+2H	+	2	2.26
90.5264	M+H	+	1	2.26
94.5460	M+2H	+	2	6.55
96.5313	M+H	+	1	1.27
98.0607	M+H	+	1	6.76
98.9846	M+H	+	1	18.26
100.9572	M+H	+	1	2.28
101.0238	M+2H	+	2	2.84
102.5328	M+H	+	1	24.77
103.5208	M+H	+	1	1.29
104.0709	M+H	+	1	1.27
104.9666	M+H-H2O	+	1	2.25
105.0038	M+H	+	1	1.27
105.0339	M+H	+	1	9.01
107.0707	M+H	+	1	10.30
112.0066	M+H	+	1	2.27
113.0348	M+H	+	1	1.34
114.0553	M+H	+	1	1.30
115.0505	M+H-H2O	+	1	1.28
115.5574	M+H	+	1	13.46
116.0709	M+H	+	1	1.83
116.9721	M+H	+	1	2.26
117.9799	M+H	+	1	2.26
118.0654	M+H	+	1	21.64
118.0865	M+H	+	1	1.29
120.9651	M+H	+	1	2.26
121.0285	M+H	+	1	19.89
124.1123	M+H	+	1	9.60
124.9558	M+H	+	1	1.14
125.9724	M+H	+	1	2.26
125.9865	M+H	+	1	2.25
126.0552	M+H	+	1	7.63
128.9861	M+H	+	1	1.30
129.0700	M+H	+	1	20.45
130.0499	M+H	+	1	1.20
130.9653	M+H	+	1	2.26
131.0492	M+H	+	1	9.08
131.0704	M+H-H2O	+	1	1.33
133.0610	M+H	+	1	1.20
137.0458	M+H	+	1	1.33
137.0459	M+H	+	1	6.68
137.0459	M+H	+	1	3.28
137.0459	M+H	+	1	6.90
138.0548	M+H	+	1	1.34

140.0106	M+H	+	1	18.26
140.0429	M+Na	+	1	1.71
141.0658	M+H	+	1	6.95
142.1227	M+H	+	1	9.59
144.1019	M+ACN+H	+	1	1.31
145.0346	M+H	+	1	1.36
146.0813	M+H-H2O	+	1	1.33
146.9966	M+H	+	1	1.30
147.1168	M+H	+	1	23.95
148.9758	M+H	+	1	2.25
149.0233	M+H	+	1	19.89
151.0966	M+H	+	1	1.34
152.1434	M+NH4	+	1	11.87
153.0406	M+H	+	1	1.34
153.0407	M+H	+	1	3.24
153.0658	M+H	+	1	1.34
154.0498	M+H	+	1	1.34
155.0539	M+H	+	1	1.27
159.0278	M+H	+	1	3.23
159.0511	M+H	+	1	1.15
160.0969	M+H	+	1	7.68
161.0118	M+K	+	1	1.31
161.0598	M+H	+	1	21.69
161.9611	M+H	+	1	1.28
163.9402	M+H	+	1	1.26
164.9481	M+H-H2O	+	1	1.26
165.0071	M+H-H2O	+	1	1.34
167.6150	M+H	+	1	16.40
169.0583	M+H	+	1	1.21
170.0326	M+H	+	1	2.13
170.1176	M+H	+	1	9.58
170.1540	M+H	+	1	11.87
171.0997	M+H	+	1	18.33
172.0457	M+ACN+H	+	1	1.28
172.1154	M+H	+	1	20.88
172.1332	M+H	+	1	9.86
172.1695	M+H	+	1	14.92
174.1125	M+H	+	1	9.91
174.1238	M+H	+	1	9.45
174.6018	M+H	+	1	14.94
174.6103	M+H	+	1	13.81
176.0553	M+H	+	1	1.36
176.0554	M+H	+	1	2.11
176.9842	M+H	+	1	1.31
177.0548	M+H	+	1	16.35
177.1638	M+H-H2O	+	1	19.12
178.1227	M+H-H2O	+	1	13.53
180.0656	M+H	+	1	9.02

180.1595	M+H	+	1	7.46
180.1748	M+NH4	+	1	13.81
181.0529	M+H	+	1	1.32
181.1224	M+H	+	1	14.86
182.5991	M+H	+	1	13.83
184.1698	M+H	+	1	12.89
185.0305	M+H	+	1	1.35
185.1148	M+H	+	1	10.34
186.1489	M+H	+	1	17.65
186.9809	M+H	+	1	2.26
188.0231	M+H-H2O	+	1	1.32
189.0871	M+NH4	+	1	1.38
190.0710	M+H+MeOH	+	1	1.36
190.5996	M+H	+	1	15.30
190.9638	M+H	+	1	1.36
191.0855	M+H	+	1	18.47
191.1042	M+H	+	1	12.38
191.1795	M+H	+	1	16.12
192.0944	M+H	+	1	19.88
194.0814	M+H	+	1	9.56
195.1018	M+H	+	1	19.30
195.1228	M+H	+	1	1.34
196.1543	M+H	+	1	1.36
197.6170	M+2H	+	2	16.38
198.0372	M+H	+	1	2.13
198.1488	M+H	+	1	11.86
198.1853	M+H	+	1	13.81
198.1854	M+H	+	1	16.48
199.0936	M+2H	+	2	16.32
199.0940	M+H	+	1	11.07
199.1694	M+H	+	1	20.42
200.2010	M+H	+	1	17.43
200.9724	M+H	+	1	1.13
203.1066	M+H	+	1	16.32
204.9795	M+H+MeOH	+	1	1.29
205.9873	M+H	+	1	1.33
206.1389	M+ACN+H	+	1	1.31
207.1743	M+H	+	1	19.83
207.1743	M+H	+	1	18.62
207.1746	M+H	+	1	19.31
208.0693	M+H	+	1	19.89
208.1308	M+Na	+	1	11.30
209.0885	M+H	+	1	19.90
209.1366	M+H	+	1	11.57
209.1384	M+H	+	1	1.29
209.1900	M+H	+	1	18.76
210.1101	M+H	+	1	9.60
210.1699	M+H	+	1	1.34

211.0940	M+H	+	1	10.70
211.1095	M+H	+	1	18.26
211.2056	M+H	+	1	23.47
212.1258	M+H	+	1	9.84
212.1645	M+H	+	1	12.86
212.6110	M+2H	+	2	19.47
213.1849	M+H	+	1	21.89
213.9797	M+H	+	1	1.30
214.1801	M+H	+	1	13.82
214.2166	M+H	+	1	18.26
215.1254	M+H	+	1	8.67
216.1959	M+H	+	1	12.86
216.9507	M+H	+	1	2.26
217.1046	M+Na	+	1	7.24
217.1434	M+H	+	1	13.55
217.1589	M+H	+	1	20.44
217.1951	M+H	+	1	20.15
218.9587	M+H-H2O	+	1	1.39
219.1743	M+H	+	1	17.25
219.1745	M+H	+	1	19.33
219.2106	M+H	+	1	23.95
219.6068	M+H	+	1	16.32
220.1178	M+H	+	1	1.35
220.1180	M+H	+	1	7.10
220.1181	M+H+MeOH	+	1	1.33
220.5964	M+H	+	1	19.89
221.1537	M+H	+	1	20.64
222.1464	M+Na	+	1	12.62
223.0965	M+H	+	1	16.35
224.1257	M+H	+	1	10.75
225.9144	M+H	+	1	1.63
226.1800	M+NH4	+	1	21.87
226.1801	M+H-H2O	+	1	13.83
227.0113	M+ACN+H	+	1	1.36
227.9952	M+H	+	1	1.28
228.1206	M+H	+	1	7.03
228.2323	M+H	+	1	19.02
230.1569	M+2H	+	2	19.92
230.9825	M+H	+	1	1.28
231.1204	M+H	+	1	7.78
231.1227	M+H	+	1	9.75
231.1591	M+H	+	1	14.50
232.1909	M+H	+	1	14.04
232.9281	M+H	+	1	2.26
233.0783	M+H	+	1	12.10
233.9821	M+H	+	1	1.43
234.1620	M+H	+	1	17.30
235.1691	M+H	+	1	19.48

235.2055	M+H	+	1	20.51
237.6605	M+2H	+	2	22.62
238.1204	M+H	+	1	12.58
238.1776	M+H	+	1	12.86
238.1984	M+H	+	1	19.76
238.2165	M+H	+	1	15.84
239.1488	M+H	+	1	7.68
239.1620	M+2H	+	2	19.91
239.2370	M+H	+	1	26.03
240.1442	2M+ACN+Na	+	1	1.39
240.1804	M+H	+	1	1.33
241.0309	M+H	+	1	1.15
242.1136	M+2H	+	2	18.69
242.1751	M+H	+	1	21.87
242.2479	M+H	+	1	19.77
243.1355	M+Na	+	1	19.11
243.1356	M+H	+	1	19.10
243.2106	M+H	+	1	23.95
244.2272	M+H	+	1	16.75
245.0767	M+H-H2O	+	1	1.28
245.1746	M+H	+	1	15.34
246.1700	M+H	+	1	17.87
246.9048	M+H	+	1	2.26
247.1693	M+H	+	1	14.84
247.1780	M+Na	+	1	15.22
248.9003	2M+Na	+	1	2.24
249.1824	M+Na	+	1	17.34
250.0797	M+Na	+	1	7.04
250.1187	M+H	+	1	17.43
250.9999	M+H	+	1	16.94
251.0468	M+H	+	1	21.14
252.1360	M+H	+	1	13.74
252.1569	M+H	+	1	12.90
253.1645	M+H	+	1	8.22
253.9093	M+H	+	1	1.60
254.1960	M+H	+	1	1.29
254.1961	M+H	+	1	7.78
254.2114	M+H	+	1	15.43
255.0288	M+ACN+H	+	1	1.34
255.1589	M+H	+	1	15.63
256.1755	M+H	+	1	7.70
256.2270	M+H	+	1	22.75
256.2635	M+H	+	1	20.29
257.2303	M+H	+	1	22.73
257.2305	M+H	+	1	16.66
258.2427	M+H	+	1	17.16
259.2055	M+H	+	1	21.11
261.1306	M+H	+	1	7.69

261.1849	M+H	+	1	20.05
261.2171	M+H	+	1	11.86
262.1436	M+H	+	1	16.31
262.1776	M+H	+	1	15.45
265.1046	M+H	+	1	15.36
267.1567	M+H	+	1	10.16
267.1721	M+H	+	1	18.27
268.2271	M+H	+	1	17.05
269.0879	M+H	+	1	1.34
269.1747	M+H	+	1	16.54
269.2112	M+H	+	1	18.17
269.2589	M+H	+	1	19.76
270.2429	M+H	+	1	17.34
270.2792	M+H	+	1	20.77
271.2267	M+H	+	1	18.23
272.2219	M+H	+	1	18.16
272.2219	M+H	+	1	15.43
272.2220	M+H	+	1	22.52
272.2583	M+H	+	1	21.98
272.2584	M+H	+	1	18.21
274.9719	M+H	+	1	1.40
275.0482	M+H	+	1	8.47
275.1101	M+H	+	1	7.87
275.1464	M+H	+	1	8.24
276.1934	M+H	+	1	16.28
277.1028	M+H+MeOH	+	1	1.36
277.2164	M+H	+	1	19.21
277.9719	M+H	+	1	1.33
278.2115	M+H	+	1	19.20
279.0934	M+H	+	1	15.10
279.1588	M+H	+	1	19.89
279.2043	M+H	+	1	13.18
279.9694	M+ACN+H	+	1	23.88
280.1676	M+H	+	1	15.77
281.1536	M+H	+	1	23.07
281.1721	M+H	+	1	18.97
281.1724	M+H	+	1	19.33
282.1464	M+H	+	1	13.83
282.2791	M+H	+	1	20.64
284.0579	M+H	+	1	11.33
284.0986	M+H	+	1	1.33
284.0988	M+H	+	1	6.93
284.2066	M+H	+	1	7.68
284.2066	M+H	+	1	1.29
284.2585	M+H	+	1	18.06
285.0829	M+H	+	1	1.33
286.2740	M+H	+	1	18.59
288.2531	M+H	+	1	16.44

289.1540	M+H	+	1	18.26
289.2483	M+H	+	1	13.83
290.2094	M+H	+	1	17.06
290.6746	M+2H	+	2	24.48
291.0699	M+H	+	1	6.68
291.1951	M+H	+	1	18.61
291.2291	M+H	+	1	19.60
292.2847	M+H	+	1	19.30
295.2266	M+H	+	1	13.77
297.0824	M+H	+	1	25.21
297.2400	M+H	+	1	19.29
298.0974	M+ACN+H	+	1	14.89
298.2223	M+H	+	1	8.22
298.3100	M+H	+	1	21.74
298.3102	M+H	+	1	22.17
299.1100	M+H	+	1	13.44
299.1853	M+H	+	1	15.29
299.2004	M+H	+	1	15.83
300.2015	M+H	+	1	8.16
304.8942	M+H	+	1	1.60
308.0887	M+H	+	1	19.17
308.0890	M+H	+	1	18.84
308.2796	M+H	+	1	17.74
309.1307	M+H	+	1	15.66
309.1828	M+H	+	1	16.36
309.2034	M+H	+	1	20.42
311.1465	M+H	+	1	13.66
311.1642	M+H	+	1	22.97
311.1855	M+H	+	1	16.37
311.2556	M+H	+	1	19.92
312.1481	M+H	+	1	14.88
312.2383	M+H	+	1	8.82
312.3262	M+H	+	1	22.77
313.2349	M+H	+	1	17.74
313.2735	M+H	+	1	20.37
314.2172	M+NH4	+	1	8.61
314.2691	M+H	+	1	20.05
315.1777	M+H	+	1	12.08
317.1151	M+H	+	1	19.90
317.2085	M+H	+	1	18.80
318.1961	M+2H	+	2	9.57
318.2064	M+H	+	1	16.33
319.1727	M+H	+	1	8.63
319.2844	M+H	+	1	14.91
321.2213	M+H	+	1	26.40
322.2715	M+H	+	1	18.04
323.1640	M+H	+	1	13.83
323.1827	M+H	+	1	16.14

327.2529	M+H	+	1	25.30
328.2329	M+H	+	1	9.18
329.2321	M+H	+	1	23.64
330.1804	M+H	+	1	18.26
331.2090	M+H	+	1	10.87
331.2629	M+H	+	1	21.65
334.1938	M+H	+	1	24.53
334.2226	M+H	+	1	15.72
334.3102	M+H	+	1	21.41
337.1046	M+H	+	1	18.22
337.2347	M+Na	+	1	16.31
338.3415	M+H	+	1	22.62
339.1541	M+H	+	1	16.32
339.2868	M+Na	+	1	21.41
341.2665	M+Na	+	1	19.19
342.1676	M+H	+	1	19.90
342.2485	M+H	+	1	8.61
343.1481	M+H	+	1	19.46
343.2841	M+H	+	1	21.71
344.2278	M+H	+	1	8.43
344.7335	M+2H	+	2	26.12
345.2265	M+H	+	1	16.39
347.3156	M+3H	+	3	20.44
348.0700	M+H	+	1	1.36
349.1832	M+H	+	1	8.44
350.2663	M+Na	+	1	19.85
353.1744	M+H	+	1	14.84
353.2086	M+H	+	1	16.30
353.2089	M+H	+	1	16.61
353.2663	M+H	+	1	19.13
353.2689	M+H	+	1	18.31
353.3047	M+2H	+	2	22.65
355.0698	M+H	+	1	26.82
355.0698	M+H	+	1	27.50
355.0701	M+H	+	1	25.95
355.2059	M+H	+	1	16.39
355.2456	M+Na	+	1	19.54
355.2822	M+Na	+	1	19.74
355.2842	M+H	+	1	25.84
356.2642	M+H	+	1	9.19
357.2997	M+H	+	1	22.19
359.0686	M+H	+	1	19.88
360.3107	M+H	+	1	16.39
361.0776	M+2H	+	2	1.21
361.3140	M+H	+	1	16.38
362.1780	M+H	+	1	21.53
363.2199	M+H	+	1	18.69
365.1357	M+H	+	1	19.78

365.2153	M+H	+	1	18.70
366.3730	M+H	+	1	22.70
367.3357	M+H	+	1	19.91
369.2609	M+Na	+	1	20.99
370.3549	M+H	+	1	24.78
371.1010	M+H	+	1	28.56
371.3155	M+H	+	1	22.64
372.3474	M+H	+	1	18.00
375.2505	M+H	+	1	19.31
376.1934	M+H	+	1	21.14
377.2144	M+H	+	1	8.78
377.2146	M+H	+	1	9.48
377.2294	M+H	+	1	22.04
378.2979	M+H	+	1	20.87
380.3371	M+H	+	1	19.13
381.3723	M+H	+	1	26.96
383.2649	M+H	+	1	21.52
383.3154	M+H	+	1	26.33
384.3475	M+H	+	1	21.83
385.2018	M+H	+	1	18.68
385.2922	M+H	+	1	18.99
388.2542	M+H	+	1	8.13
390.2093	M+H	+	1	22.54
393.2094	M+H	+	1	8.76
393.2200	M+H	+	1	21.53
393.2975	M+H	+	1	23.58
394.3529	M+H	+	1	19.67
395.2202	M+H	+	1	21.53
397.2921	M+H	+	1	22.10
397.4038	M+H	+	1	26.57
399.2111	M+H	+	1	22.06
399.2507	M+H	+	1	18.77
399.2719	M+Na	+	1	19.44
399.3080	M+Na	+	1	19.67
399.3584	M+H	+	1	20.51
399.7642	M+2H	+	2	22.65
404.1378	M+H	+	1	19.89
404.3133	M+Na	+	1	21.15
405.3401	M+H	+	1	16.35
406.3291	M+H	+	1	21.82
407.1837	M+H	+	1	18.69
407.3323	M+H	+	1	22.55
409.3677	M+H	+	1	24.29
410.2226	M+H	+	1	21.52
411.0936	M+Na	+	1	19.49
413.2895	M+H	+	1	20.12
415.1905	2M+H	+	1	16.32
415.2326	M+H	+	1	14.70

416.3523	M+H	+	1	23.04
417.3366	M+H	+	1	20.23
419.2773	M+H	+	1	19.22
420.1689	M+H	+	1	16.32
420.7298	M+2H	+	2	26.72
421.2327	M+H	+	1	18.76
423.2517	M+H	+	1	22.55
424.4513	M+H	+	1	25.73
425.2143	M+H	+	1	21.02
425.2868	M+H	+	1	21.63
427.3059	M+H	+	1	20.95
428.3368	M+H	+	1	20.83
429.3188	M+H	+	1	18.88
429.3190	M+Na	+	1	19.03
431.3842	2M+H	+	1	21.88
432.2804	M+H	+	1	9.07
433.3314	M+H	+	1	24.87
434.3605	M+H	+	1	22.70
436.3998	M+H	+	1	21.49
437.2356	M+H	+	1	9.07
437.3740	M+H	+	1	18.04
438.2176	M+H	+	1	21.54
439.3182	M+H	+	1	22.00
441.3215	M+H	+	1	21.13
441.3936	M+H	+	1	24.31
445.1200	M+H	+	1	27.80
445.1201	2M+H	+	1	25.95
445.1202	M+H	+	1	27.17
445.1204	M+H	+	1	25.95
445.1205	M+H	+	1	24.37
453.4667	M+H	+	1	27.57
455.3131	M+H	+	1	24.86
455.4094	M+H	+	1	24.96
457.3293	M+H	+	1	20.88
459.3022	M+H	+	1	24.00
459.3557	M+H	+	1	18.03
467.4096	M+H	+	1	27.22
468.3899	M+H	+	1	18.95
475.4146	M+H	+	1	25.59
476.2121	M+H	+	1	22.01
476.3068	M+H	+	1	8.88
476.3069	M+H	+	1	9.27
477.4666	M+H	+	1	27.21
480.2780	M+H	+	1	10.53
481.2619	M+H	+	1	9.28
481.2843	M+H	+	1	24.01
482.4571	M+H	+	1	24.90
482.8614	M+2H	+	2	20.38

483.4410	M+H	+	1	24.96
485.1129	M+H	+	1	21.00
493.1455	M+H	+	1	21.53
495.4772	M+H	+	1	26.74
497.4572	M+H	+	1	25.85
503.1076	M+H	+	1	26.12
503.2966	M+H	+	1	17.50
508.8569	2M+Na	+	1	1.60
509.5298	M+H	+	1	28.86
512.4161	M+H	+	1	18.87
519.1392	M+H	+	1	27.16
520.3326	M+H	+	1	9.65
520.4365	M+H	+	1	26.03
522.3850	M+H	+	1	25.30
525.2880	M+H	+	1	9.32
525.2883	M+H	+	1	9.53
527.1924	M+H	+	1	22.77
528.3085	M+H	+	1	17.92
531.4048	M+H	+	1	23.53
532.4111	M+H	+	1	24.61
535.4699	M+H	+	1	26.53
536.1656	M+H	+	1	26.11
537.5359	M+H	+	1	25.10
538.1448	M+NH4	+	1	23.99
538.3140	M+H	+	1	18.20
541.1208	M+H	+	1	26.09
549.4125	M+H	+	1	25.84
552.3300	M+H	+	1	18.41
553.3906	M+H	+	1	24.52
557.0944	M+H	+	1	26.11
559.1317	M+H	+	1	22.25
559.5181	M+H	+	1	24.99
560.4711	M+H	+	1	26.09
564.3592	M+H	+	1	9.12
565.5672	M+H	+	1	25.62
569.3840	M+Na	+	1	25.30
575.5040	M+H	+	1	31.28
577.1270	M+H	+	1	26.82
577.4441	M+H	+	1	26.34
579.2929	M+H	+	1	19.88
579.4960	M+H	+	1	26.33
593.1577	M+H	+	1	27.79
593.1583	M+H	+	1	23.40
593.1584	M+H	+	1	29.45
593.5983	M+H	+	1	26.18
607.5662	M+H	+	1	27.37
608.3856	M+H	+	1	9.93
610.1843	M+H	+	1	26.83

612.1818	M+H	+	1	26.92
622.6137	M+H	+	1	26.30
628.3749	M+H	+	1	27.59
628.5002	M+NH4	+	1	19.87
628.8602	M+H	+	1	1.60
631.1140	M+H	+	1	26.83
633.1501	M+H	+	1	23.40
643.5275	M+H	+	1	26.56
644.4952	M+NH4	+	1	18.66
646.5266	M+H	+	1	26.74
651.1454	M+H	+	1	27.49
663.4539	M+H	+	1	26.72
668.1770	M+H	+	1	27.86
684.2031	M+NH4	+	1	27.48
685.4357	M+H	+	1	26.70
686.2005	M+H	+	1	27.48
688.5215	M+NH4	+	1	18.59
698.2551	M+H	+	1	28.52
707.1691	M+H	+	1	24.37
722.4907	M+H	+	1	26.72
741.1942	M+H	+	1	25.25
742.1967	M+H	+	1	25.22
752.5590	M+H	+	1	24.89
753.5666	M+H	+	1	27.48
756.6351	M+H	+	1	26.60
772.2736	M+H	+	1	29.56
772.6296	M+H	+	1	23.69
774.5433	M+H	+	1	24.27
776.2327	M+H	+	1	25.21
781.1874	M+H	+	1	25.22
782.6503	M+H	+	1	24.09
789.6678	M+H	+	1	21.86
812.6970	M+H	+	1	25.39
816.2159	M+H	+	1	25.95
846.2922	M+H	+	1	30.82
850.2513	M+H	+	1	25.95
851.2526	M+H	+	1	25.94
852.2510	M+H	+	1	26.03
855.2052	M+H	+	1	25.95
871.7355	M+H	+	1	28.80
874.2509	M+H	+	1	29.40
882.4929	M+H	+	1	26.25
883.4951	M+H	+	1	26.20
889.2329	M+H	+	1	26.58
929.2236	M+H	+	1	26.58
963.2515	M+H	+	1	27.16
965.2501	M+H	+	1	27.17

Table S2. Validation metrics of PLS-DA models.

**Parameter of PLS-DA model**	**Tissue:** Brain		
Class	BALB/c	C57BL/6	CD1
Data preprocessing	Log_{10}, mean center		
N	5	5	5
Number of LV	3		
RMSEC	0.091	0.132	0.127
RMSECV	0.120	0.193	0.179
	**Tissue:** Liver		
Class	BALB/c	C57BL/6	CD1
Data preprocessing	Log_{10}, mean center		
N	5	5	5
Number of LV	3		
RMSEC	0.133	0.128	0.141
RMSECV	0.174	0.186	0.225
	**Tissue:** Kidney		
Class	BALB/c	C57BL/6	CD1
Data preprocessing	Log_{10}, mean center		
N	5	5	5
Number of LV	4		
RMSEC	0.067	0.093	0.105
RMSECV	0.160	0.218	0.209
	**Tissue:** Muscle		
Class	BALB/c	C57BL/6	CD1
Data preprocessing	Log_{10}, mean center		
N	5	5	5
Number of LV	2		
RMSEC	0.092	0.216	0.184
RMSECV	0.142	0.279	0.259

Table S3. Metabolic pathways and involved metabolites differentiating tissues of C57BL/6, BALB/c and CD1 mouse strains.

**Pathway name**	**Compound**
Pantothenate and CoA biosynthesis	Pantothenic acid<br>Valine<br>Uracil
Aminoacyl-tRNA biosynthesis	Asparagine<br>Valine<br>Proline
Valine, leucine and isoleucine biosynthesis	Valine
Beta-alanine metabolism	Uracil
Glutathione metabolism	Pyroglutamic acid
Cysteine and methionine metabolism	Cystine
Valine, leucine and isoleucine degradation	Valine
Tryptophan metabolism	2-aminobenzoic acid
Arginine and proline metabolism	Proline
Pyrimidine metabolism	2′- deoxycytidine<br>Uracil
Alanine, aspartate and glutamate metabolism	N-acetylaspartic acid<br>Asparagine
Purine metabolism	Xanthine<br>Adenosine monophosphate (AMP)<br>5-hydroxyisourate
Terpenoid backbone biosynthesis	Mevalonic acid
Sphingolipid metabolism	Ceramide (d40:1)

Table S4. Isotope pattern matching analysis of differential metabolites using the ChemSpider database.

Compound	Molecular weight	ChemSpider results	Full match	Δ mass [ppm]	Matched/ missed isotopes	Sfit [%]	Pattern Coverage [%]
Uracil	112.0276	3	1	2.42	3/0	81	99.61
Proline*	115.0636	8	1	2.23	2/0	91	99.05
Valine*	117.0792	14	1	2.14	5/0	65	99.87
Pyroglutamic acid*	129.0427	11	1	0.43	5/0	86	100
Asparagine	132.0536	8	1	0.81	2/0	92	98.62
2-aminobenzoic acid	137.0476	15	1	-0.45	5/0	60	100
Mevalonic acid	148.0737	28	1	0.83	1/0	100	93.06
Xanthine*	152.0334	3	1	-0.01	2/0	84	98.13
Allantoin	158.0439	2	1	-0.80	2/0	54	98.03
N-acetylvaline	159.0896	18	1	0.57	2/0	84	98.86
Tetrahydrodipicolinate	171.0532	4	1	0.09	2/0	97	98.56
N-acetylaspartic acid	175.0481	4	1	-0.06	4/0	81	100
5-hydroxyisourate	184.0233	2	2	0.42	2/0	61	97.86
Pantothenic acid	219.1107	2	1	0	2/0	86	98.59
2'-deoxycytidine	227.0905	3	3	-0.44	4/0	81	100
Cystine	240.0237	3	1	-0.69	2/0	90	92.84
2-methylbutyrylcarnitine	245.1627	4	1	0.06	2/0	95	98.59
Inosine*	268.0807	6	1	-0.42	4/0	67	99.90
N-tridecanoylglycine	271.2147	1	1	-0.58	2/0	93	98.59
Oleamide*	281.2718	3	1	-0.29	4/0	66	100
8-hydroxydeoxyguanosine	283.0916	6	1	-0.41	3/0	65	98.75
Ethyl eicosapentaenoic acid	330.2557	14	2	-0.58	2/0	87	97.43
Adenosine monophosphate*	347.0629	9	2	-0.49	3/0	78	99.85
Ceramide (d40:1)	621.6063	2	1	0.43	4/0	70	99.87

Sfit – Spectral similarity score between theoretical and measured isotope pattern in %

* Fragmentation spectrum confirmed with mzCloud database.
